# Repair of Dissecting Aortic Aneurysm in Post-LVAD Patient

**DOI:** 10.14797/mdcvj.1363

**Published:** 2024-06-06

**Authors:** Sanjay Chaubey, Parag Kale, Dan Meyer, Aldo Rafael

**Affiliations:** 1 Baylor Scott & White, Baylor University Medical Center, Dallas, Texas, US; 2 Baylor University Medical Center, US; 3 Annette C and Harold C. Simmons Transplant Institute, Baylor Scott & White Research Institute, Dallas, Texas, US

**Keywords:** CF-LVAD, aortic dissection, aortic aneurysm

## Abstract

Left ventricular assist devices (LVAD) are frequently used in the management of end-stage heart failure, especially given the limited availability of donor hearts. The latest HeartMate 3 LVAD delivers non-physiological continuous flow (CF), although the impact on the aorta is not well established. We highlight a case of aortic aneurysm formation complicated by dissection formation that necessitated high-risk re-operative surgery in a patient post CF-LVAD.

## Introduction

Ventricular assist devices are an important technological development for patients with decompensated end-stage heart failure. The effect of nonphysiological flow generated by continuous flow left ventricular assist device (CF-LVAD) on the aorta remains poorly understood. We report a case in which the patient developed a dissection in an ascending aortic aneurysm with aneurysmal dilation of the descending aorta. The aortic dilation developed following LVAD insertion, necessitating a difficult redo surgery and endovascular management of the descending aorta.

## Case Presentation

A 61-year-old female with stage D heart failure underwent a Heartmate 3 (Abbott Cardiovascular) LVAD placement 6 years prior. The patient had a family history of aortic disease affecting her mother and two brothers; however, her ascending and descending aorta measured 3.6 and 3.4 cm, respectively, at the time of LVAD insertion.

**Figure 1:**
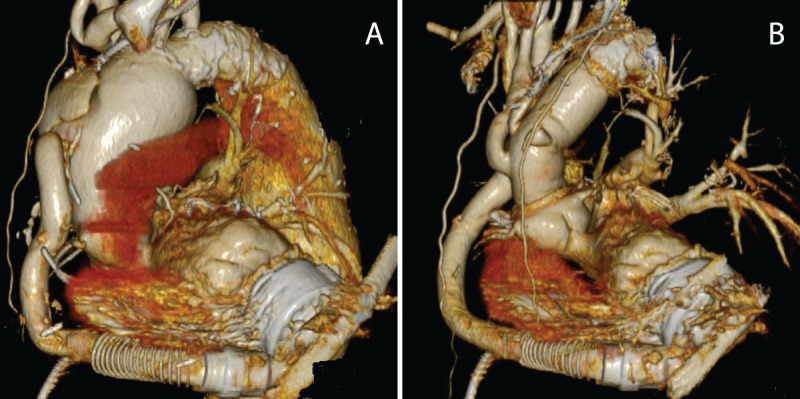
**(A)** The heart is seen with the left ventricular assist device (LVAD) in-situ. The ascending aorta is dilated and attached with the anastomosed LVAD outflow graft. **(B)** The ascending aorta is replaced.

Follow-up computerized tomography (CT) scans showed the development of an aneurysmal dilatation of the ascending aorta, which remained stable in size. However, the follow-up CT in 2023 showed a dissection in the ascending aortic aneurysm, which was now 7.1 cm in size (Figure 1A). The dissection extended from the cannula-aorta anastomosis to the distal ascending aorta and involved the proximal aortic arch. The patient also developed aneurysmal dilatation of her descending aorta of 7.2 cm in size. Following a departmental meeting, the decision was made to proceed with high-risk surgery to correct the ascending and arch dilation followed by endovascular management of the descending aorta.

## Surgery

The groin was exposed and a left subcostal incision was made, starting from the midline to gain access to the outflow graft. A section of the bend relief was removed and the outflow graft exposed. This approach is routinely undertaken at our institution and allows for access for arterial cannulation should the need arise. Access was gained to the axillary artery and femoral vein to establish cardiopulmonary bypass. The LVAD was turned off and the outflow graft was clamped. After median sternotomy, the patient was cooled to 24°C for hypothermic arrest, the aorta was clamped, and the heart was arrested with antegrade cardioplegia. The ascending aorta was opened and resected, and the outflow graft was detached from the aorta with further cardioplegia given via the coronary arteries. Deep hypothermic circulatory arrest was achieved, and selective antegrade cerebral perfusion was administered via the right axillary artery. The proximal descending aorta was dilated and calcified. Using an open distal approach, the aortic arch was further cut proximal to the left common carotid artery to allow for distal reconstruction in a suitable area of the aortic arch, being careful of the left recurrent laryngeal nerve. A Dacron graft with side branches and felt reinforcement was chosen, and a distal anastomosis was performed with re-implantation of the brachiocephalic artery and left common carotid artery. The aortic cross-clamp was re-applied more proximally on the Dacron graft, and full bypass and re-warming was reestablished. The proximal aortic anastamosis was then performed and the aorta subsequently de-aired. Using a side-biting aortic clamp, the LVAD outflow graft was attached to the proximal ascending aorta (Figure 1B). Six months later, the patient was admitted for endovascular repair of her descending aortic aneurysm.

## Discussion

Given the shortage of organs available for transplant, LVADs are being used more frequently. However, our case highlights the importance of patient selection. Although our patient had a family history of aneurysm formation, she did not have any such problem at the time of her LVAD insertion. Thus, the patient did not undergo genetic testing. However, over the course of 6 years, she developed an aneurysmal dilatation of both her ascending and descending aorta, possibly related to the CF-LVAD device itself.

There is a growing body of evidence that some of the common CF-LVAD-related complications, such as gastrointestinal bleeding, may arise from vascular adaptation to changes in physiological blood flow during CF-LVAD.^1^ Ambardekar et al.^2^ reported that CF-LVADs lead to a profound down-regulation in extracellular matrix degradation and found that the aorta had become more fibrotic. Segura et al.^3^ also studied aortic tissue before and after LVAD implantation and found medial degeneration, elastic fiber fragmentation, and medial fibrosis. Both studies discussed the weakening of the aorta over time with CF-LVAD implantation. The combination of family history of aneurysm formation and loss of pulsatility from CF-LVAD may have contributed to aneurysm formation with subsequent dissection of the aorta given time and size.

Such surgery is rarely reported in the literature. However, there are implications from this case report for surgeons undertaking CF-LVAD insertion—namely, patients need to be counselled about the possibility of redo surgery from potential future aortic wall pathology and/or consider undergoing surgical intervention on the aorta at the time of LVAD insertion. Indeed, Lee et al.^4^ reported their decision to perform hemiarch replacement for severe aortic atherosclerosis at the time of LVAD implantation. Thus, the potential for future surgery on the aorta would need to be factored into the planning, especially if a CF-LVAD is being planned as destination therapy to avoid complex redo surgery.

## Conclusion

The use of CF-LVAD in heart failure management plays an important role. However, we continue to learn about its long-term effects. Due to the potential for aortic wall weakening post CF-LVAD insertion, patients and surgeons must consider the possible need for future aortic surgery even in patients with no aortic problems at the time of insertion. The patient must be informed of potential risks from their past medial or family history and counselled about undergoing surgical intervention at the time of insertion to circumvent the need for complex re-do surgery in the future, especially in patients receiving CF-LVAD as destination therapy.
